# Association of certain biochemical parameters related to bone cycle with genotype in MPS IIIB patients

**DOI:** 10.55730/1300-0144.5973

**Published:** 2025-01-07

**Authors:** Seda GÖKKURT, İrem PEKER EYÜBOĞLU, Banu NUR GÜZEL, Ercan MIHÇI, Ayşe ÖZER, Mustafa AKKİPRİK

**Affiliations:** 1 Division of Medical Biology and Genetics, Department of Health Sciences, Faculty of Medicine, Marmara University, İstanbul, Turkiye; 2 Division of Pediatric Genetics, Department of Pediatrics, Faculty of Medicine, Akdeniz University, Antalya, Turkiye

**Keywords:** Mucopolysaccharidoses, NAGLU, NGS, osteoporosis

## Abstract

**Background/aim:**

The aims of this study are to investigate the genotype–phenotype correlation in Sanfilippo type B (MPS IIIB) patients in terms of bone formation/resorption parameters and to determine the release/inhibition of biomarkers accompanying osteoporosis.

**Materials and methods:**

Plasma levels of osteoprotegerin (OPG), matrix metalloproteinases (MMP2 and MMP9), tissue inhibitors of metalloproteinase (TIMP1 and TIMP2) and cathepsin K were examined using the ELISA method for a MPS IIIB patient group and a control group. At the same time, mutations in the NAGLU gene causing the disease were identified by whole exome sequencing, and their correlation with biochemical parameters was investigated.

**Results:**

The enzyme analysis results showed that MMP2, MMP9, TIMP1, and TIMP2 were significantly high in the study group, while cathepsin K was low. OPG levels were similar between the two groups. The genetic analysis of patients with MPS IIIB was performed by sequencing all exons and exon–intron junction regions of the NAGLU gene using a next-generation sequencing (NGS) system. In this way, variations were detected qualitatively with high read depths. The analyses found that only two patients had a previously pathogenically defined alteration. In addition, the impact assessment analyses detected alterations with a modifying effect on protein structure.

**Conclusion:**

The genetic analysis results indicate the need to consider a variation classified as benign in the OMIM database as pathogenic because the variations found in the patients (p.Arg737Gly and p.Trp103Cys) have somehow altered enzyme activity. The mutation p.Trp103Cys, a novel NAGLU gene mutation in the first exon, was detected in one patient; additionally, SIFT and PolyPhen analyses confirmed it as damaging. Further functional analyses of this variation should be conducted to gather more comprehensive information.

## 1. Introduction

Sanfilippo type B (MPS IIIB) is an autosomal recessive disease in the group of lysosomal storage diseases known as mucopolysaccharidoses (MPS), caused by mutations in the gene that produces the enzyme required to break down a specific sugar called heparan sulfate [1,2]. There are four types of MPS III, named A, B, C, and D, which have very few clinical differences between them. The differences arise from mutations in different regions of the gene that enable the production of specific enzymes. According to Di Natale and Peltekian one in every 70,000 babies born worldwide may have one type of MPS III [3,4].

Genetic diseases can lead to osteoporosis due to both primary and secondary defects. There are many mechanisms responsible for the etiopathogenesis of secondary defects. In metabolic diseases, such as Fabry disease, malnutrition, malabsorption, chronic kidney failure, and decreased physical activity are responsible for osteoporosis. Osteoporosis is also observed in Gaucher patients due to skeletal involvement. Typical skeletal manifestations in MPS patients are disorganization of the growth plate and shortened bones, indicating a problem in endochondral ossification, and MPS III patients also experience osteoporosis as a secondary condition [5]. Studies have shown that in predicting bone loss and fracture risk, biochemical parameters related to the bone cycle provide more useful information than measurements of bone mineral density (BMD) [6].

Two key parameters provide information on bone turnover or osteoporosis. One is cathepsin K, an important enzyme for bone resorption that is produced by osteoclasts. High glycosaminoglycan (GAG) concentration has been reported to have an inhibitory effect on the collagenolytic activity of cathepsin K during bone growth and development. It regulates the synthesis and release of cathepsin K and may be a potential target for the treatment of bone turnover or osteoporosis [7]. The OPG/RANKL ratio is another important determinant of bone mass and plays an important role in the regulation of bone metabolism [8]. TRACP-5b has been recognized as a marker for osteoclast activity and it is increased in osteoporosis. Osteoblast-mediated MMPases have been shown to play an active role in the initiation of bone resorption by degrading the unmineralized osteoid layer on the bone surface and allowing osteoclasts to attach to the mineralized matrix [9]. Matrix metalloproteinase MMP9 originates from infiltrating neutrophils, and gelatinase has been shown to play a role in rheumatoid arthritis and synovitis. Therefore, MMP2 and MMP9 are expected to increase in osteoporosis activities. However, in MPS III patients, MMP9 serum activity and protein levels decrease significantly, gelatinolytic activity decreases, and MMP2 activity increases significantly [10].

In MPS IIIB disease, common genetic variations have been identified by sequencing all exons and exon–intron junctions of the NAGLU gene using a next-generation sequencing system to compare the variations with the phenotype. By evaluating variations with high sequencing depths, performing all statistical analyses, and examining the relationships between genotype and biochemical parameters and clinical effects, the genotype–phenotype correlation can be determined [11,12]. The goal is to provide guidance for the clinical use of bone turnover parameters in MPS IIIB patients by correlating these parameters with genotype and identifying the molecular mechanisms responsible for osteoporosis associated with metabolic diseases. The study aims to identify common responsible molecular mechanisms and provide a prospective idea for targeted therapy in line with pathophysiology.

## 2. Materials and methods

This study was conducted as a collaboration between the Department of Pediatric Genetics at the Faculty of Medicine of Akdeniz University and the Department of Medical Biology and Genetics at Marmara University, with MPS IIIB patients diagnosed at the former, and their genetic analyses performed at the latter. The study protocol was approved by the Ethics Committee of Akdeniz University and was conducted in accordance with the principles of patient information management (No. 70904504/94). It was conducted as retrospective study, including eight MPS IIIB patients (male and female) and 10 control group subjects of the same age and gender range. The average patient age was 9.2 years, and the clinical characteristics of the patients are presented in Table 1. Patients 2 and 3 and patients 7 and 8 are siblings.

Inclusion criteria for the study were having clinical symptoms and findings of MPS IIIB and the differential diagnosis of the disease being clarified by enzyme analysis compared to other MPS III subtypes. Exclusion criteria for the study were previously having undergone hematopoietic stem cell transplantation and having any other known systemic disease detected by medical history, examination, or laboratory investigations. The differential diagnosis of MPS IIB compared to the other subtypes was clarified by genetic test or enzyme analysis showing decreased GALNS enzyme activity in fibroblasts or leukocytes. Patients with clinical symptoms and signs of MPS IIIB were included in the study.

Values for OPG (Bendermed), MMP2 and MMP9, tissue inhibitors of metalloproteinase TIMP1 (Life Tech) and TIMP2 (Calbichem), OPG (Bendermed), cathepsin K (UscnLife) values were examined using ELISA kits. The Marmara University Scientific Research Projects Unit provided support for the enzyme analysis kits used in the study (Project no. SAG-C-YLP-100914-0322).

Genomic DNA was isolated using the QIAamp DNA purification kit according to the manufacturer’s instructions. Statistical analyses were performed using MedCalc v.12.7.7. Since this disease belongs to a very rare disease group, a regression analysis was not performed for the statistical parameters. However, the relationships and clinical effects between genotype and biochemical parameters were investigated.

An Illumina Nextera XT kit was used for amplicon sequencing procedures, and its sequencing protocol can be used for sequencing up to 96 indexed polymerase chain reaction (PCR) products. DNA fragmentation and adapter sequence additions within the protocol were carried out using the contents of the DNA Library Preparation Kit and Index Kit to make the template DNA compatible with the sequencing device and analyzed.

The fragmentation and adapter addition stages of the samples were performed using a process called tagmentation in hard shell plate wells. After the PCR products were cleaned, they were diluted to a concentration of 2 ng/μL with the Qubit device and solutions and then brought to a concentration of 0.2 ng/μL and loaded into the wells. After the DNA was tagmented, it was subjected to a PCR reaction with index primers and the Nextera PCR Mastermix. With 12 cycles of PCR, index sequences were added to the ends of the tagmented DNA and each sample was barcoded with a separate index.

After the PCR, all samples were pooled together by equalizing to 2 mmol using a buffer solution. The pooled DNA library was prepared for loading into the NextSeq 500 by denaturation and dilution. Finally, the sequencing reaction was completed by loading it into a 600 μL cartridge.

## 3. Results

ELISA kits were used to analyze all biochemical parameters in duplicate. Based on the enzyme analysis results shown in Table 2, MMP2 (p = 0.01), MMP9 (p = 0.043), TIMP1 (p = 0.014), and TIMP2 (p = 0.005) were significantly lower in the control group, while cathepsin K was lower in the MPS IIIB group (p = 0.002). OPG levels were similar between the two groups.

Data on the correlation between the study and control groups are presented in Tables 3 and 4, respectively. Values for OPG, MMP2, MMP9, TIMP1, TIMP2 and cathepsin K were examined.

In the study group, the ELISA parameters did not show a statistically significant correlation with each other. However, the MMP2 parameter was found to be statistically significantly lower in the control group compared to the study group.

There is a high correlation between the MMP9 and TIMP1 parameters in the control group (r = 0.745, p = 0.013), as well as a high correlation between the MMP2 and cathepsin K ELISA parameters (r = 0.770, p = 0.009).

The results of the genetic and biomarker analyses of the MPS IIIB patients are shown in Table 5. According to the OMIM database, only one change (p.Arg234Gly) has been reported to have a pathogenic effect based on base changes. No clinical effect has been reported for the other homozygous changes (p.Arg737Gly and p.Trp103Cys), while the variants c.531+50G>C and p.Ser141Ser are labeled as benign in the OMIM database. The p.Trp103Cys variant is a novel variant found in the first domain of the gene. The codon 737 region change was observed in six patients. This change indicates a change in the alpha-helical region of the NAGLU protein [13,14].

## 4. Discussion

MPS IIIB is a rare and devastating childhood-onset lysosomal storage disease caused by complete loss of function of the lysosomal hydrolase α-N-acetylglucosaminidase. The lack of a functional enzyme in MPS IIIB patients leads to the progressive accumulation of heparan sulfate throughout the body and triggers a cascade of neuroinflammatory and other biochemical processes, ultimately resulting in severe mental impairment and early death in adolescence or young adulthood [15].

MPS IIIB patients suffer from developmental regression, bone malformation, organomegaly, gastrointestinal distress, and profound neurological deficits. Enzyme replacement therapies (ERTs) SBC-103 (uses a recombinant form of the N-acetyl-α-D-glucosaminidase (NAGLU) enzyme) and AX250 (also known as tralesinidase alfa) are still being investigated as treatments for MPS IIIB. Despite human trials of ERTs for MPS IIIB, there is currently no FDA approved treatment and few palliative options. The major concerns of ERT and gene therapy for the treatment of bone malformation are the inadequate biodistribution of the missing enzyme, N-acetyl-α-glucosaminidase (NAGLU) and that the skeleton is a poorly hit target tissue in ERT and gene therapy. Each of the four known human types of MPS III (A, B, C, and D) is usually regarded as having mild bone manifestations, yet all remain poorly characterized.

In this study, OPG levels were found to be similar between the study and control groups, while cathepsin K levels were significantly lower in the MPS IIIB group. It has been noted that a high concentration of glycosaminoglycans inhibits the collagenolytic activity of cathepsin K during bone growth and development [16]. MMPs have been shown to play an active role in initiating bone resorption by degrading the mineralized osteoid layer on the bone surface to allow osteoclasts to attach to the mineralized matrix [17–19], which can help explain why MMP9 and MMP2 were found to be significantly lower in the control group. Consistent with these values, the TIMP1 and TIMP2 levels were also found to be lower in the control group compared to the study group.

Given the fact that MMPs seem to be involved in numerous neuroinflammatory diseases, and an overproduction of these enzymes is coupled with a disruption of the blood–brain barrier [20], MMP2 and MMP9 might represent suitable biomarkers to indicate central nervous system involvement. One rat study on BMD suggested that there is a negative correlation between MMP9 levels and BMD [21]. Another study found that serum OPG levels in MPS patients were linked to elevated levels of RANKL and AXIN1, and increased levels of the growth factors Flt3L and SCF were observed. Increased osteoclastogenesis could be the reason for this elevation as osteoclasts are of hematopoietic origin and hematopoietic stem cells (HSCs) give rise to myeloid progenitors upon stimulation [22,23]. GAG storage, which induces a complex sequence of molecular changes leading to inflammation, synovial hyperplasia, and cartilage apoptosis, is assumed to play a major role in joint and bone pathologies in MPS diseases. The latest mouse model study showed that mid-diaphyseal BMC was significantly increased in MPS IIIB mice compared to controls, with males showing a 15.9% increase and females an 11.1% increase before epiphyseal closure. Metaphyseal BMC was also elevated, with males showing a 110.7% increase and females a 47.9% increase compared to the controls. They stated that the MPS IIIB mice exhibited significant measurable changes in femoral radiological and biomechanical properties, varying by sex and age, and proposed the model’s utility for preclinical therapeutic research targeting orthopedic complications in MPS IIIB. [24].

The accumulation of undigested GAGs in the lysosomes of connective tissue cells and chondrocytes is responsible for musculoskeletal abnormalities commonly observed in almost all MPS subtypes. Since the results of GAG electrophoresis in urea can vary between laboratories, even in buffer solutions and conditions, it was not possible to take a standardized measurement between the patients in this study. All of the patients had very low enzyme values, making it possible to make general clinical comparisons between patients.

Recently, upregulation of CTSA, CTSH, and CTSZ has been detected through transcriptomic and proteomic analyses in a rat model of spinal cord injury [25]. The cysteine protease cathepsin K, which has long been known as a molecular marker of differentiated osteoclasts and is directly involved in the degradation of bone matrix proteins, plays a crucial role in skeletal pathologies frequently observed in MPS and in other lysosomal storage diseases (LSDs) [26,27].

The enzyme analysis results of all these studies revealed that MMP2, MMP9, TIMP1, and TIMP2 were low and cathepsin K was high in MPS III patients, and that the patients had low bone density and increased osteoporosis risk. For this reason, it has been recommended that MPS III patients should be followed up regularly in terms of bone health and osteoporosis risk [28,29]. That outcome was not supported by this study, but that may be due to the low number of patients included.

In support of this study, the contribution of cathepsins to pathophysiological changes in MPS IIIB patients has been examined, and they were shown to be involved in the onset and progression of neuropathology and skeletal disorders in MPSs. Affected patients may benefit from treatment with cathepsin-targeting drugs [30]. Therefore, it will be important to test the efficacy of a new generation of highly selective cathepsin inhibitors in MPSs. These drugs could be used alone or in combination with existing therapeutic approaches to improve the quality and duration of life of these patients. However, more research is needed into the active role of different cathepsins in the pathophysiology of MPSs so that they can be recognized as key players in the fight against such incurable diseases.

In one recent study, three proteins were found to be uniquely upregulated in NAGLU knockout mouse cerebrospinal fluid (CSF), spanning a wide range of functions and signaling processes. TNFRSF11B, normally involved in bone homeostasis, can be expressed in brain glial and neuronal cells in certain ischemic stress conditions, indicating a role in brain injury following inflammation. [31]. TNFRSF11B may be one of the molecules that should be looked at regarding bone turnover in MPS patients.

Animal and human studies investigating the treatment effects of anti-inflammatory drugs such as pentosan polysulphate on skeletal pathologies in MPS IIIB are being undertaken. In addition to inflammation, many studies with various MPS animal models have reported early abnormalities of chondrocyte organization in the growth plate and architecture of the cortical bone, which could be a trigger for abnormal bone modeling and remodeling leading to secondary hip deformities in MPS III [32].

As a result of the genetic analysis done in this study, the p.Arg234Gly change, which had previously been identified as pathogenic, was found in two patients. The impact assessment analysis showed that this change had a protein structure-altering effect according to both SIFT and PolyPhen algorithms.

The p.Arg737Gly change, found in six patients, has been classified as damaging by SIFT but as benign by PolyPhen. Considering the clinical results of the patients, this change might affect enzyme activity. To determine the exact effect of this variation, further functional analysis is needed with larger patient groups. The p.Trp103Cys change in the first exon, found in only one patient, had not been previously identified in the dbSNP global variation database. This result shows that this is a novel variation first detect in this study. In addition, the SIFT and PolyPhen analyses confirmed the variation as damaging. Accordingly, a more comprehensive study can be carried out in the future by conducting functional analyses of the variation. The pathogenic changes confirmed by genetic analysis and variations believed to be associated with the disease show that genetic structure has serious effects on the disease. Future projects based on the results obtained in this study will be able to confirm candidate variations and diagnose MPS IIIB patients before symptoms become clear. In addition, it will be much easier to distinguish MPS IIIB from other diseases that have similar phenotypes.

Regarding the genetic analysis results, it is necessary to consider a variation classified as benign in the OMIM database as pathogenic because the variations (p.Arg737Gly and p.Trp103Cys) found in the patients have somehow altered enzyme activity.

Inconsistent with the results of many studies conducted on bone turnover and osteoporosis risk in MPS patients, this study supports the finding of high levels of MMP2, MMP9, TIMP1, TIMP2, and low levels of cathepsin K in MPS IIIB patients, indicating that their bone density is low and their risk of bone turnover or osteoporosis is increased [12,23–33]. One 2020 study integrated diverse expression quantitative trait loci and splicing quantitative trait loci data with several powerful GWAS datasets to identify novel candidate genes associated with osteoporosis. In total, they detected 88 genes significantly associated with trabecular BMD or fracture through expression or ribonucleic acid splicing. NAGLU is one of the genes that is in the protein–protein interaction network for osteoporosis. [34]. Therefore, regular monitoring of bone health and osteoporosis risk is recommended for MPS III patients.

Obtaining a correlation with a very low number of patients was the main limiting factor of this study. The rareness of the disease makes it difficult to reach high patient numbers. With further translational analyses or studies on enzyme–enzyme interactions, more insight could be gained into this disease and its accompanying pathophysiological effects.

Another limitation of this study is that it only included bone turnover biomarkers, whereas many other parameters, such as bone fractures, should also be considered when evaluating osteoporosis. Nonetheless, future researchers should take note of the finding that cathepsin K levels were lower in the MPS IIIB group. This observation may support the idea that acidification contributes to the dissolution of the mineral component of the bone matrix, thereby exposing the organic component to degradation by cathepsin K.

## Figures and Tables

**Table 1:** Patient clinical characteristics.

Patient	Diagnosis age (years)	Sex	Dysmorphic face	Scoliosis dysostosis	Phenotype
1	11	Female	Yes	Yes	Severe
2	5	Male	Yes		Mild–moderate
3	8	Male	Yes	Yes	Mild
4	5	Male	Yes		Mild
5	5	Male	Yes		Mild
6	5	Female	Yes	Yes	Moderate
7	17	Female	Yes	Yes	Severe
8	9	Male	Yes	Yes	Severe

**Table 2:** Comparison between the study and control groups.

	MMP9 (pg/mL)	TIMP1 (pg/mL)	OPG (pg/mL)	TIMP2 (pg/mL)	Cathepsin K (ng/mL)	MMP2 (pg/mL)
Study group	162	185.6	117.8	14.5	1804.4	29
Control group	97	143.1	80.1	11.1	3475.6	15.7
p	0.043	0.014	0.161	0.005	0.002	0.01

MPS = mucopolysaccharidoses, MMP = matrix metalloproteinases, TIMP = tissue inhibitor of metalloproteinases, and OPG = osteoprotegerin.

**Table 3:** Correlations between ELISA parameters for the study group.

Spearman’s rank correlation coefficient	MMP9 human ELISA	TIMP1 human ELISA	OPG human ELISA	TIMP2 human ELISA	Cathepsin K human ELISA	MMP2 human ELISA
MMP9 human ELISA	–	−0.107, 0.819*	−0.536, 0.215*	0.071, 0.879*	0.107, 0.819*	0.321, 0.482*
TIMP1 human ELISA	−0.107, 0.819*	–	−0607, 0.148*	0.321, 0.482*	0.107, 0.819*	0.286, 0.535*
OPG human ELISA	−0.536, 0.215*	−0.607, 0.148*	–	−0.214, 0.645*	0.071, 0.879*	−0.750, 0.052*
TIMP2 human ELISA	0.071, 0.879*	0.321, 0.482*	−0.214, 0.645	–	−0.286, 0.535*	−0.357, 0.432*
Cathepsin K human ELISA	0.107, 0.819*	0.107, 0.819*	0.071, 0.879	−0.286, 0.535*	–	−0.071, 0.879*
MMP2 human ELISA	0.321, 0.482*	0.286, 0.535*	−0.750, 0.052*	−0.286, 0.535*	−0.071, 0.879*	–

MPS = mucopolysaccharidoses, MMP = matrix metalloproteinases, TIMP = tissue inhibitor of metalloproteinases, OPG = osteoprotegerin, and

* = p.

**Table 4:** Correlations between ELISA parameters for the control group.

Spearman’s rank correlation coefficient	MMP9 human ELISA	TIMP1 human ELISA	OPG human ELISA	TIMP2 human ELISA	Cathepsin K human ELISA	MMP2 human ELISA
MMP9 human ELISA	–	0.745, 0.013*	0.359, 0.309*	0.333, 0.381*	0.309, 0.385*	0.491, 0.150*
TIMP1 human ELISA	0.745, 0.013*	–	0.103, 0.776*	0.567, 0.112*	0.042, 0.907*	0.503, 0.138*
OPG human ELISA	0.359, 0.309*	0.103, 0.776*	–	0.251, 0.515*	0.134, 0.713*	0.340, 0.336*
TIMP2 human ELISA	0.333, 0.381*	0.567, 0.112*	0.251, 0.515*	–	−0.083, 0.831*	0.267, 0.488*
Cathepsin K human ELISA	0.309, 0.385*	0.042, 0.907*	0.134, 0.716*	−0.083, 0.831*	–	0.770, 0.009*
MMP2 human ELISA	0.491, 0.150*	0.503, 0.138*	0.340, 0.336*	0.267, 0.488*	0.770, 0.009*	–

MPS = mucopolysaccharidoses, MMP = matrix metalloproteinases, TIMP = tissue inhibitor of metalloproteinases, OPG = osteoprotegerin, and

* = p.

**Table 5:** NAGLU variant analysis results.

Patient	HGVS consequence	dbSNP ID	ClinVar	SIFT	PolyPhen	MMP9 (pg/mL)	TIMP1 (pg/mL)	OPG (pg/mL)	TIMP2 (pg/mL)	Cathepsin K (ng/mL)	MMP2 (pg/mL)	Phenotype
1	p.Ser141Ser	rs659497	Benign	TOLERATED	–	346.63	153.99	76.81	16.24	659.45	31.37	Severe
	c.531+50G>C	rs2071046	Benign	–	–							
	p.Arg737Gly	rs86312	Benign	Deleterious (0.02)	Benign (0.154)							
2	p.Ser141Ser	rs659497	Benign	TOLERATED	–	116.47	151.25	291.88	13.27	3361	24.86	Mild–moderate
	p.Arg234Gly	rs104894601	Pathogenic	Deleterious (0.04)	Probably damaging (0.958)							
3	p.Ser141Ser	rs659497	Benign	TOLERATED	–	97.67	193.18	146.36	15.7	1560.7	9.81	Mild
	p.Arg234Gly	rs104894601	Pathogenic	Deleterious (0.04)	Probably damaging (0.958)							
4	p.Ser141Ser	rs659497	Benign	TOLERATED	–	229.49	199.45	47.26	12.97	2448.88	42.67	Mild
	c.531+50G>C	rs2071046	Benign	–	–							
	p.Arg737Gly	rs86312	Benign	Deleterious (0.02)	Benign (0.154)							
5	p.Ser141Ser	rs659497	Benign	TOLERATED	–	103.4	181.42	122.63	11.03	737.37	33.2	Mild
	c.531+50G>C	rs2071046	Benign	–	–							
	p.Arg737Gly	rs86312	Benign	Deleterious (0.02)	Benign (0.154)							
6	p.Trp103Cys	–	–	Deleterious (0)	Probably damaging (1)	302.5	140.23	66.23	16.52	702.34	30.4	Moderate
	p.Ser141Ser	rs659497	Benign	TOLERATED	–							
	c.531+50G>C	rs2071046	Benign	–	–							
	p.Arg737Gly	rs86312	Benign	Deleterious (0.02)	Benign (0.154)							
7	p.Ser141Ser	rs659497	Benign	TOLERATED	–	111.57	211.99	58.21	17.42	1829.55	31.53	Severe
	c.531+50G>C	rs2071046	Benign	–	–							
	p.Arg737Gly	rs86312	Benign	Deleterious (0.02)	Benign (0.154)							
8	p.Ser141Ser	rs659497	Benign	TOLERATED	–	128.73	208.07	81.55	14.98	2033.89	29.34	Severe
	c.531+50G>C	rs2071046	Benign	–	–							
	p.Arg737Gly	rs86312	Benign	Deleterious (0.02)	Benign (0.154)							

## Data Availability

All data generated and analyzed during this study are included in this article. Further enquiries can be directed to the corresponding author.
